# The global contribution of invasive vertebrate eradication as a key island restoration tool

**DOI:** 10.1038/s41598-022-14982-5

**Published:** 2022-08-10

**Authors:** Dena R. Spatz, Nick D. Holmes, David J. Will, Stella Hein, Zachary T. Carter, Rachel M. Fewster, Bradford Keitt, Piero Genovesi, Araceli Samaniego, Donald A. Croll, Bernie R. Tershy, James C. Russell

**Affiliations:** 1 Pacific Rim Conservation, Honolulu, HI USA; 2 The Nature Conservancy, Santa Cruz, CA USA; 3 Island Conservation, Santa Cruz, CA USA; 4 University of Auckland, Auckland, New Zealand; 5 American Bird Conservancy, Santa Cruz, CA USA; 6 Institute for Environmental Protection and Research (ISPRA), Rome, Italy; 7 IUCN SSC Invasive Species Specialist Group, Rome, Italy; 8 Manaaki Whenua—Landcare Research, Auckland, New Zealand; 9 UC Santa Cruz, Santa Cruz, CA USA

**Keywords:** Environmental impact, Conservation biology, Invasive species

## Abstract

Islands are global hotspots for biodiversity and extinction, representing ~ 5% of Earth’s land area alongside 40% of globally threatened vertebrates and 61% of global extinctions since the 1500s. Invasive species are the primary driver of native biodiversity loss on islands, though eradication of invasive species from islands has been effective at halting or reversing these trends. A global compendium of this conservation tool is essential for scaling best-practices and enabling innovations to maximize biodiversity outcomes. Here, we synthesize over 100 years of invasive vertebrate eradications from islands, comprising 1550 eradication attempts on 998 islands, with an 88% success rate. We show a significant growth in eradication activity since the 1980s, primarily driven by rodent eradications. The annual number of eradications on islands peaked in the mid-2000s, but the annual area treated continues to rise dramatically. This trend reflects increases in removal efficacy and project complexity, generating increased conservation gains. Our synthesis demonstrates the collective contribution of national interventions towards global biodiversity outcomes. Further investment in invasive vertebrate eradications from islands will expand biodiversity conservation while strengthening biodiversity resilience to climate change and creating co-benefits for human societies.

## Introduction

Islands are a focal point in the global biodiversity crisis. The disproportionately higher rates of endemism, endangerment and extinction on islands when compared to continents^1–3^ makes islands a priority target for conserving biodiversity. Invasive alien species (hereafter, “invasive species”), especially invasive terrestrial mammals, are the primary driver of native biodiversity loss on islands^4^, and are also linked to declines in human health and livelihoods, including billions of dollars (US$) in island-based economic losses^5–7^. Permanent eradication of invasive species populations from islands has become a foundational conservation action^8,9^, resulting in significant progress towards reducing global extinctions^10^, and thus contributing to partial achievement of Aichi Target 9 between 2011 and 2020^11^. Benefits to native species on islands from the eradication of invasive species have repeatedly accrued in terrestrial habitats and are evident in near-shore habitats, facilitating complimentary restoration actions such as reforestation and conservation translocations^9^. Over 80% of the world’s archipelagos are thought to have invasive rodents^12^, thus a vast number of islands are likely invaded. However, highly threatened (IUCN Red List Critically Endangered or Endangered) native species currently persist on less than 1% of the world’s islands, many of which are invaded yet can benefit from invasive species eradication^2,13^. Invasive species eradications on islands present a significant and tangible opportunity to reverse global extinction trends, with ensuing co-benefits of improved human health and livelihoods and strengthened biodiversity resilience to climate change^14,15^.

Synthesizing the actions that improve global biodiversity outcomes provides key insights for future conservation decision-making^16^. For invasive species eradications, a lack of data-driven synthesis of interventions hinders integration of evidence into conservation decision-making and best practice^17^. This is mostly driven by fragmented reporting, particularly underreporting of failures, and challenges in accessing gray literature and expert knowledge^16^. Previous reviews of eradications attempted to address this problem by providing descriptions of operational methods associated with success and failure^18,19^, yet these reviews were not designed to systematically track eradication events over time. The development of a global database in 2010 provided a necessary standardized framework to systematically document the methods and outcomes of eradications implemented worldwide^20,21^. The database has since undergone a decade of systematic data reviews with rigorous data quality controls^22^, resulting in a unique compendium of global invasive vertebrate eradications that can be analyzed to provide an understanding of the global contribution of invasive vertebrate eradications as a key island restoration tool.

Here we provide the first data-driven synthesis of all reported eradication activities of invasive vertebrates on islands worldwide before 2020. We assess global trends in eradication events, invasive species targeted, success rates of eradication efforts, and geographies where these efforts took place. We also conduct a novel timeline analysis to examine trends through time. Our approach provides a model for collating and interpreting data on the utility of global conservation tools, which is advantageous to scale biodiversity gains^23,24^. Our findings advance conservation by demonstrating the collective contribution of national interventions towards global biodiversity outcomes, and validate eradication as an effective and routine biodiversity conservation tool. These results serve as a guide for data-driven conservation decisions, including policy and funding that support effective invasive species eradications on islands.

## Results

### Eradication events and success rates

**Figure 1:**
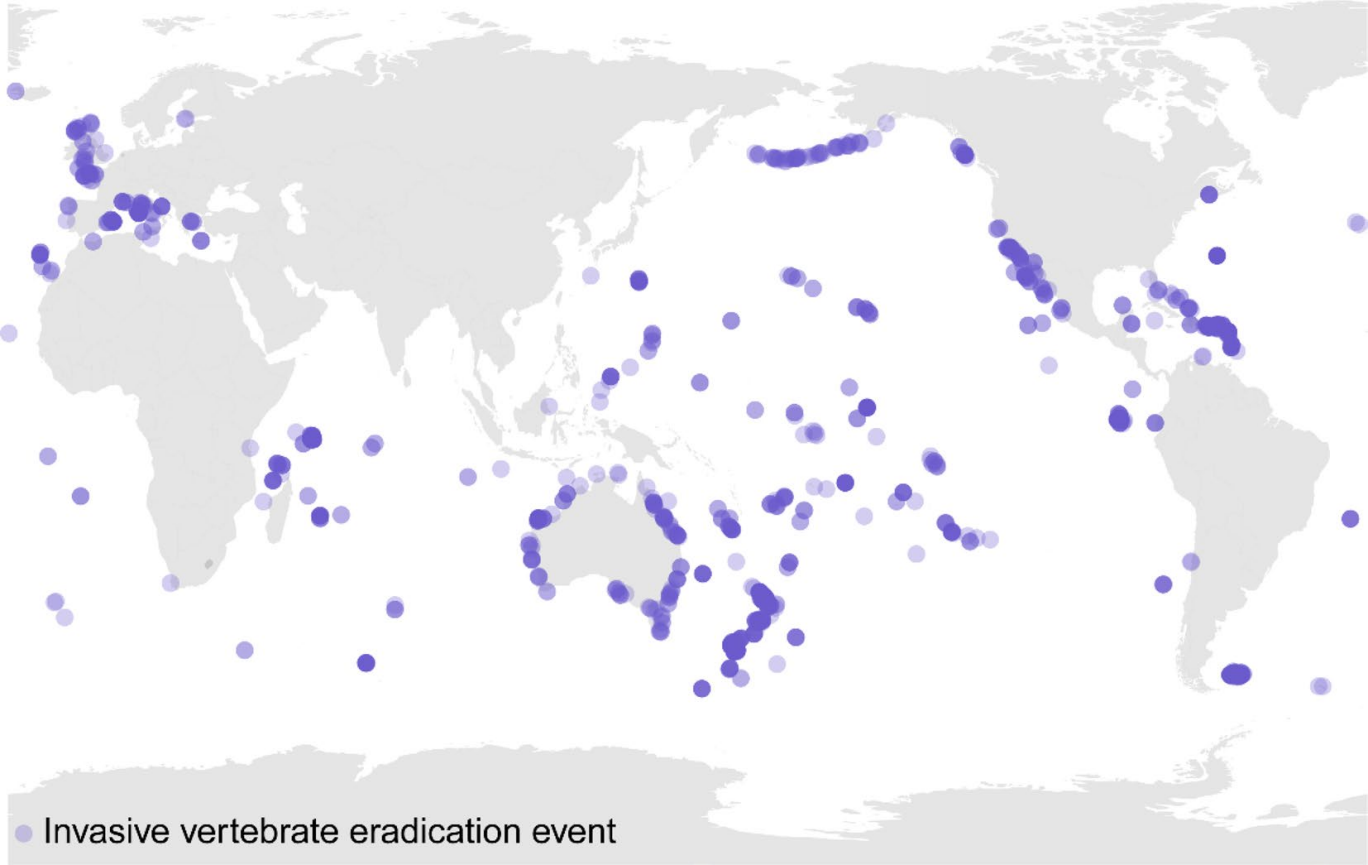
The island locations of all invasive vertebrate eradication events, 1872–2019. Each purple dot represents an eradication event on an island, with darker dots indicating higher numbers of eradication events.

A total of 1550 eradication events were documented on 998 islands since 1872 (Fig. 1, Table 1). Eradication targets were invasive mammals (97.2% of events) on 990 islands, birds (2.5%) on 30 islands, amphibians (0.3%) on three islands, and reptiles (0.1%) on one island. Of these events, 88% (1081 of 1227 completed attempts without subsequent reinvasion) were successful, while 12% failed. Success rates for invasive mammals and birds were 88% and 82%, respectively, ranging from 73 to 96% by invasive taxa. Most eradications targeted *Rattus* spp. (820 events, 53%, of which 99% targeted *R. rattus, R. norvegicus* and *R. exulans*) with 95% of 520 successful events achieved using toxicants. Other common target species included ungulates (12%; 70% targeted *Capra* hircus) and *Felis* spp. (8%;99% targeted *Felis catus*) with 54% and 87% of successful events achieved using hunting and trapping, respectively. Sixty-two percent of all invasive bird events targeted *Acridotheres tristis* (Common Myna; flying) or *Gallirallus australis* (Weka; non-flying). Among successful bird eradications, the primary methods used were hunting for flying birds (64%), and toxicants for non-flying birds (50%).

**Table 1:** Invasive vertebrate eradication targets, methods, and outcomes, 1872–2019, ordered by the total number of events per group.

Invasive targets				Outcomes				Area treated* (by 1000 ha)					Island human habitation		Primary eradication method		
	Species targeted	Islands targeted	Total # events	Successful events	Reinvaded events	Failed events	Success rate (%)	Median area (ha)	Mean area (ha)	5th percentile area (ha)	95th percentile area (ha)	Total area (ha)	Uninhabited islands	Inhabited islands	Primary method	Primary method (n)	Primary method (%)
Rats	4	666	820	545	135	74	88.0	0.01	0.17	0.0	2.57	94.86	496	49	Tx	520	95
Ungulates	12	147	185	150	4	13	92.0	0.51	10.80	0.009	255.09	1619.3	103	47	H	130	87
Cats	2	105	117	80	5	12	87.0	0.26	2.28	0.026	36.50	182.38	51	29	Tr	43	54
Mice	1	86	91	51	7	19	72.9	0.06	0.50	0.004	7.67	25.42	43	8	Tx	50	98
Rabbits and hares	3	80	89	74	1	7	91.4	0.09	0.41	0.007	5.24	30.16	63	11	Tx	41	55
Dogs and foxes	4	64	69	59	2	7	89.4	3.50	9.36	0.035	66.66	552.31	54	5	Tr	36	61
Stoats, weasels, mink and mongoose	5	60	66	49	7	2	96.1	0.08	5.42	0.007	127.05	265.56	39	10	Tr	40	82
Other mammals	11	32	39	25	0	5	83.3	0.08	3.36	0.001	51.81	84.04	20	5	Tx	11	44
Pigs	1	29	30	24	1	2	92.3	1.07	8.08	0.085	52.67	193.96	16	8	H	21	88
Flying birds	10	16	24	14	0	3	82.4	0.15	0.46	0.028	2.71	6.39	1	13	H	9	64
Non-flying birds	4	15	15	8	4	2	80.0	0.16	4.99	0.006	24.18	39.93	6	2	Tx	4	50
Frogs and toads	1	3	4	1	1	0	NA	0.01	0.01	0.007	0.01	.007	0	1	O	1	100
Lizards	1	1	1	1	0	0	NA	0.22	0.22	0.224	0.22	.224	1	0	Tr	1	100
Total	59	998	1550	1081	167	146	88.1	–	–	–	–	–	893	188		907	–

Rats include species from the *Rattus* genus, mice include only *Mus musculus.* Outcomes include success rates, calculated as Successful Events/(Successful + Failed Events). Event details (area treated, island habitation, and primary method) are summarized for successful events only. Primary method codes: Tx (Toxicant), Tr (Trapping), H (Hunting), O (Other).

*Islands counted more than once if they had multiple success eradication events (e.g., rat and cat eradication).

### Island biogeographies

Eradication events were located at latitudes between 65° N (Flatey Island, Iceland) and 54° S (Macquarie Island, Australia), and primarily in tropical (385 islands, 38.6%) and temperate (309 islands, 34.4%) climactic zones (Supplementary Data). Three-hundred eradication events (19% of events) were on 135 islands inhabited by humans (13.5% of islands), with a success rate of 82% (188 successful events; Table 1). Eradications occurred on 52% of islands within Small Island Developing States and in a total of 38 countries, comprising 25% of the world’s countries with islands, of which 41% were island nations. Country incomes were primarily classified as high (57%) and upper middle (30%). Eighty percent of eradications were carried out by eight countries: New Zealand (24.1%), Australia (12.6%), United Kingdom (12.3%), United States (11.1%), France (8.6%), Mexico (4.0%), The Seychelles (3.9%) and Ecuador (3.7%). Twenty-one percent of all eradication events occurred on a territory within the national jurisdiction of the United Kingdom, France, United States, or Australia, comprising 47% of eradications conducted by these countries.

### Temporal trends

**Figure 2:**
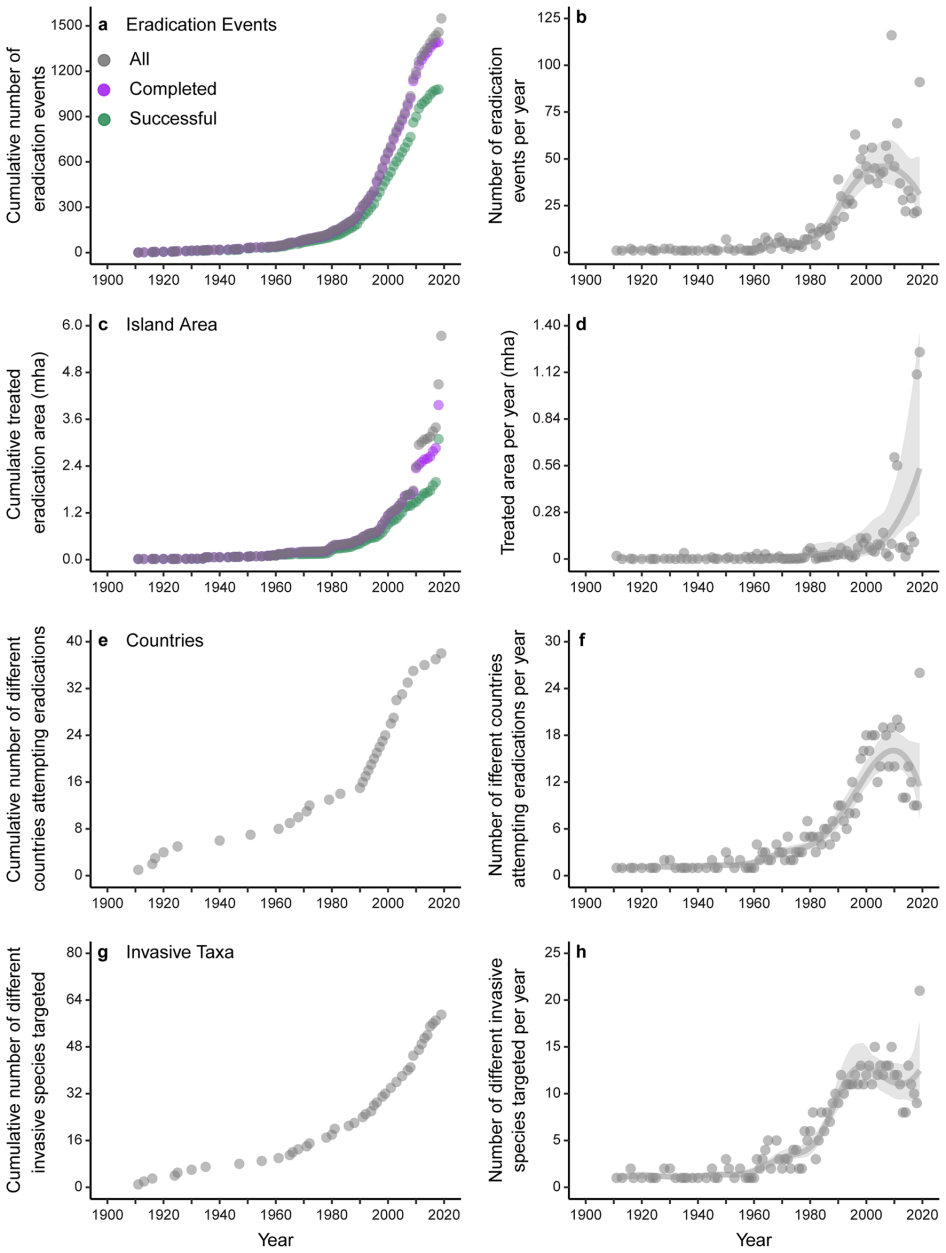
Trends in invasive vertebrate eradications over time, 1900–2019. Cumulative numbers and annual numbers are presented in the left and right columns, respectively. Panels represent the number of eradication events (**a**, **b**), treated island area (**c**, **d**), number of countries implementing eradications (**e**, **f**), number of invasive species targeted (**g**, **h**). Panels **a** and **c** display the three modes of nested data: all events, completed events, and successful events; the remaining panels display all eradication events.

**Figure 3:**
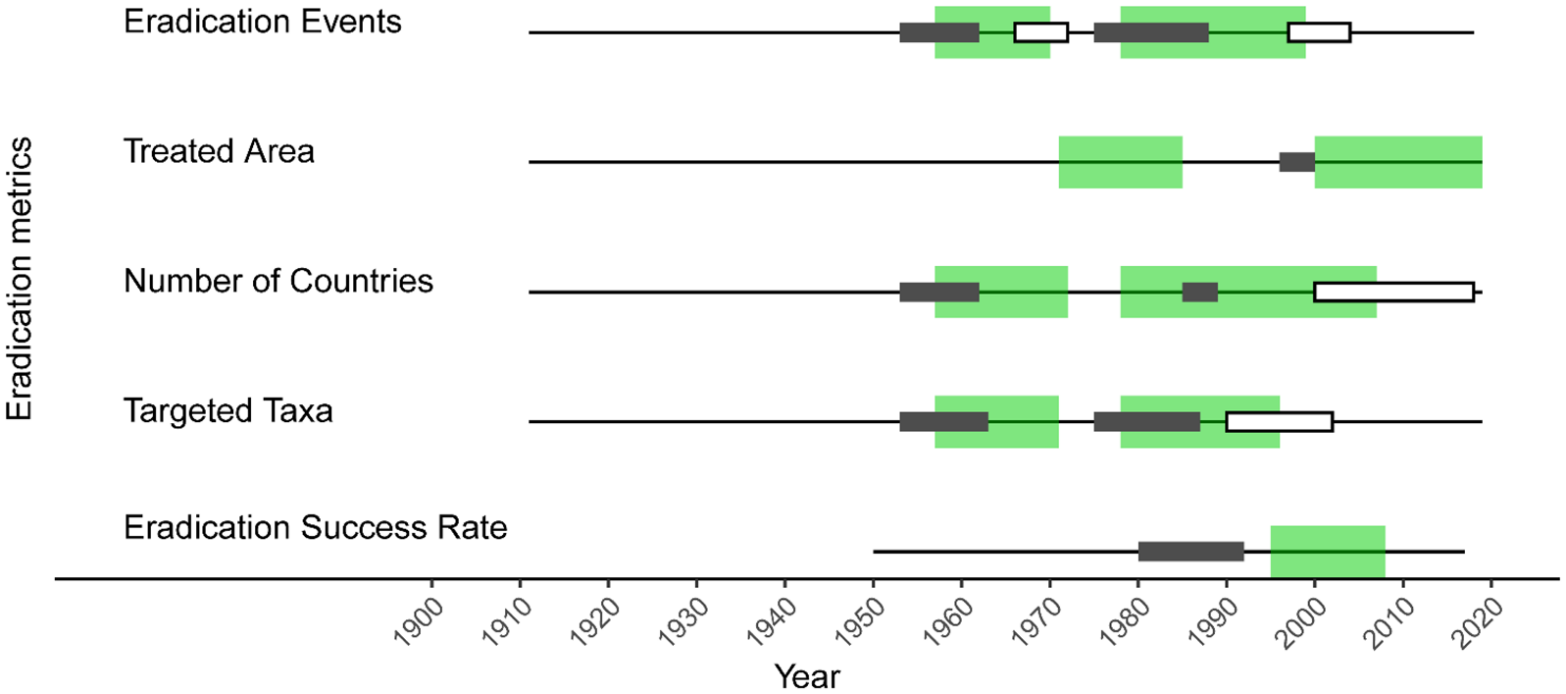
Eradication timeline analysis, 1900–2019. Significant periods of change over time are shown for the eradication metrics displayed as curves in Fig. 2 (annual number of eradication events (2b), treated island area (2d), countries implementing eradications (2f), invasive taxa targeted for eradication (2h)) and Fig. 4 (eradication success rate). Significant periods are indicated when lasting five or more years: positive gradient (i.e., increasing slope, wide green bar), positive curvature (i.e., acceleration; narrow dark bar), and negative curvature (i.e., deceleration; narrow white bar). There were no periods of significant negative gradient (i.e., decreasing slope) for any of the analyzed metrics.

**Figure 4:**
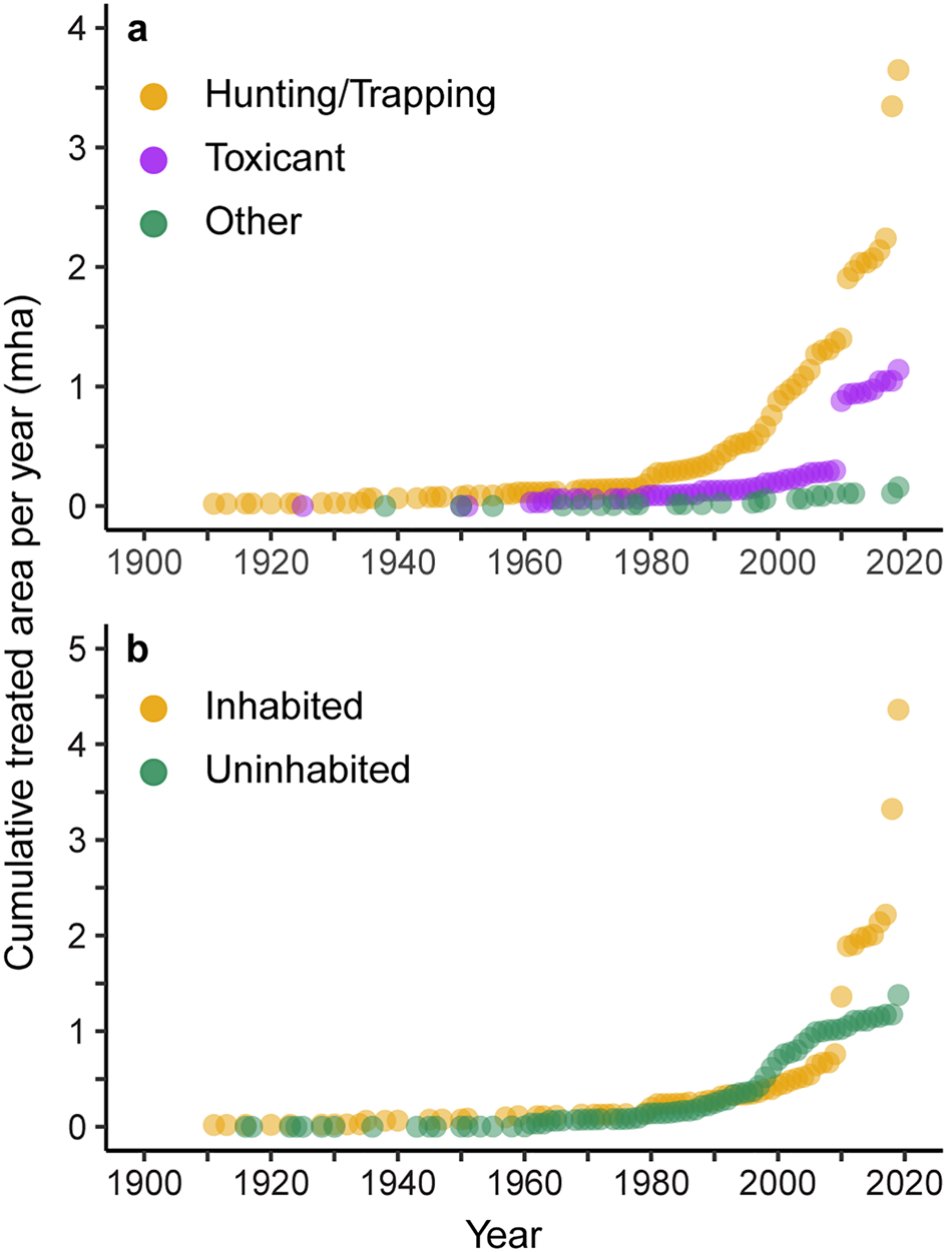
Eradication method trends by area, 1900–2019. Cumulative island areas targeted annually with the application of each major eradication method (**a**) and the human habitation status on those islands (**b**).

**Figure 5:**
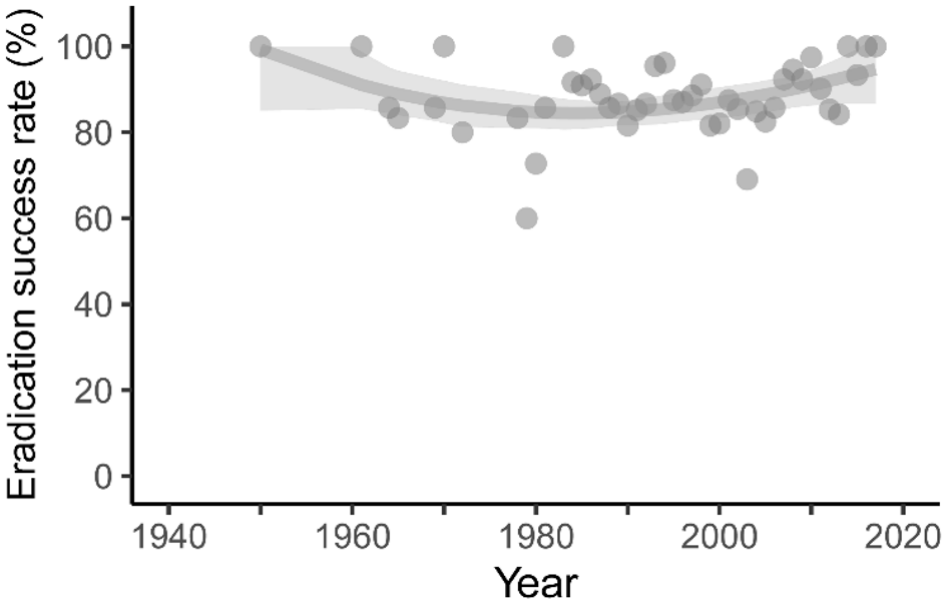
Annual success rates of completed eradication events, 1900–2019. Each point represents a success rate calculated for all events in a year with ≥ 5 eradication events (calculated as successful events/[successful + failed events]). Gray shading represents 95% confidence intervals.

Most eradication events occurred after 1950 (1524 events, 98.3%, Fig. 2). There were two periods of significant growth in eradication activity that were synchronous among three different metrics: number of eradication events, number of countries where eradications were implemented, and the number of invasive taxa targeted per year (Figs. 2a,b,e–h, 3). Each period was marked by an acceleration in the trajectories of these three metrics, followed by a significantly positive rate of increase. The first growth period started in the 1950s, when ungulate eradications by hunting were predominant (Fig. 4a; Supplementary Fig. 1) and lasted until the 1970s. The second period started in the 1980s, coinciding with widespread uptake of toxicant methods for eradicating rodents, and lasted into the 2000s. Eradication success rate across all events was high and changed infrequently over time (mean = 87.7%, lower CI = 81–87%, upper CI = 87–100%; Fig. 5), although there was a small significant improvement from the 1980s to the mid-2000s (Fig. 3).

The annual number of eradication events, countries implementing eradications, and invasive taxa targeted slowed between the late 1990s or mid- 2000s (Fig. 2a,b,e–h, Fig. 3). In the 2010s, there were apparent declines in the annual numbers of eradication events and countries alongside a small increase in the annual number of invasive taxa targeted, but these changes were not significant. During this decade, annual eradication events also appeared to decline among some of the common early adopter nations, such as New Zealand, United States, and Seychelles, with the overall contribution from countries outside of these nations becoming more prominent (Supplementary Fig. 2).

The annual area of islands treated increased exponentially from the 1960s onwards (Fig. 2d,e, Supplementary Fig. 3) with island area increasing annually across all invasive taxa (Supplementary Fig. 4). There was significant growth in treated area in the years following each of the two growth periods described above, indicating that from the 1980s onwards there was an alternation between growth in the number of eradication events and growth in the size of eradication area treated (Fig. 3). Continued exponential growth in island area through the 2000s and 2010s coincided with an increase in invasive species eradications on human inhabited islands (Fig. 4b), suggesting a shift in global eradication efforts towards fewer but more complex operations. Variability in treated area was considerably higher before the mid-1980s than afterwards (Supplementary Fig. 3), suggesting that earlier decades did include ambitious high-area targets, but at a lower frequency than later decades.

## Discussion

In general, the current pace of conservation action is insufficient to meet global biodiversity targets and reverse current extinction trajectories^25^. Invasive species are a primary driver of native biodiversity loss worldwide, especially on islands where extinctions have been concentrated^4,26^. The imperative to mitigate impacts caused by invasive species is included in the draft post-2020 global biodiversity framework, which includes a target aimed at eradicating invasive species in key priority areas such as islands^27^. Islands offer some of the greatest opportunities for biodiversity conservation through invasive species eradications^9^, including strengthened ecosystem resilience and improved human livelihoods^15^.

This synthesis describes the growth in implementation and adoption of invasive vertebrate eradications, collectively contributing to the global Convention on Biological Diversity post-2020 framework^25^ and UN 2030 Agenda for Sustainable Development^15^. The results of the synthesis reveal an acceleration of this local conservation action and its collective contribution to biodiversity conservation on a global scale^9^. The steady and relatively high rate of successful eradications at 88%, across invasive taxa and geographies, demonstrates the utility and broad applicability of this conservation intervention (Fig. 5). This outcome mimics rates identified for optimal learning^28^, where low failure rates provide learning opportunities that drive successes on subsequent attempts and can lead to ground-breaking conservation innovations^29,30^.

Standardized global datasets that characterize success rates of conservation interventions are rare^24^. Our results demonstrate that invasive species eradication is an effective and routine means to recover threatened biodiversity and meet national and global conservation targets in an era of accelerating biodiversity loss^8,9,11^. Using the Database of Island Invasive Species Eradications^22^ and a novel timeline analysis, we have provided a model for collating and interpreting data on the utility of conservation tools that are applied globally, which is urgently needed to scale biodiversity conservation^23,24^. This dataset is also an authoritative source for tracking and measuring progress towards achieving the United Nation’s conservation targets, in addition to its sustainable development goals^15^. Beyond these benefits, eradication science that emerges from such datasets can provide cross-disciplinary guidance to other sectors^31^. This is particularly relevant in this current time period as island nations are considering how to leverage their isolation to protect their communities from global threats^32^.

### Temporal trends reveal scalable innovations of eradication tools

Eradications of invasive vertebrates on islands initially focused on ungulates, rabbits, cats, and pigs using hunting and trapping methods. While implementation of these successful methods are increasing, in particular on large islands, they do not approach the magnitude of rodent eradication events, which is consistent given the harmful impacts of rodents on island biodiversity^12^. The development of toxicant-based removal methods in the 1970s facilitated the widespread increase in rodent eradication activity in the 1980s^33^. During the 1990s, innovations in aerial toxicant broadcast methods further enabled operations, particularly on larger islands^34^, and which were adopted by countries worldwide while maintaining a relatively high success rate over time.

Island eradication patterns appeared to shift in the 2000s and 2010s, with a decline in the annual number of eradication events coinciding with an increase in the targeted island land area of those events. This indicated a transition to ambitious operations on large islands, including more operations on human-inhabited islands. Large island size and inhabitation dramatically increase project complexity and cost, and can carry an increased risk of failure and non-target impacts, resulting in longer timeframes for approval and implementation^35^. However, these islands are also likely to have higher biodiversity value^36^, indicating the potential for greater conservation gains. Our results showed nearly 16% of eradications occurred on islands with globally threatened vertebrates, a slight increase since last reported in 2017 at 11%^2^. In addition to biodiversity impacts, invasive species on inhabited islands negatively interact with human livelihoods, thus these eradications have a greater likelihood to contribute to sustainable development goals^15^.

This synthesis also revealed an increase in the diversity of invasive species targets over time, as techniques were adapted to different invasive vertebrate groups (Fig. 2g,h, Supplementary Figs. 1, 4). Further, the growing application of eradication strategies to remove birds (Supplementary Discussion), and development of methods for harmful invasive invertebrates (e.g., ants^37^), indicates the transferability across taxa of developed technical and operational methods. The success of existing eradication methods, and the continued persistence of invasive species on islands, can be expected to drive more eradication project innovations, notably with respect to stakeholder and community engagement, species-specific toxicants, genetic biocontrols, and rapid detection methods^21,35,38^. Improvements in eradication technologies are still necessary for the tool to reach its potential^11,39^, and government and private sector investment remains essential to support the continued growth and efficacy of eradications as a globally significant conservation tool^40,41^.

### National-level contributions to global island restoration outcomes

Eradication efforts were primarily driven by just eight countries responsible for 80% of all eradications: New Zealand, Australia, France, United Kingdom, United States, Mexico, Seychelles, and Ecuador. Nearly a quarter of these eradications took place on 28 territories of United Kingdom, France, United States and Australia in the Atlantic, Pacific and Indian Oceans, highlighting the resource availability from higher income nations in driving technique development and implementation of projects globally^42^. Notably, eradication rates appear to decline in France, United States and Australia in the 2010s, yet these countries still hold threatened biodiversity on islands with invasive species, highlighting an unrealized need and opportunity^2,40^. Conversely, apparent declines in other countries may reflect national progress in eradicating invasives from islands^43^. For example, in the Seychelles, successful invasive vertebrate eradications on over 20 islands improved biodiversity outcomes and provided a foundation for achieving subsequent conservation actions, including conservation translocations of 20 native species to restored islands^44^. In New Zealand, where successful eradications have been achieved on over 100 islands, ambitions are refocusing towards larger, inhabited islands, and mainland-focused operations, including innovations in localized baiting and trapping techniques, the installation of predator-proof fencing, and progress towards a nationwide Predator Free 2050 campaign^45–47^.

Elsewhere, the rate of eradication efforts is increasing. Before the 1950s, fewer than 10 countries conducted invasive species eradications, while at the start of the 2020s there were 38. These 38 countries represent a quarter of the world’s coastal countries (e.g., Italy, Canada), and nearly half of all island nations (e.g., Japan, Kiribati), on a diverse array of islands, including islands in Small Island Developing States, where globally threatened biodiversity and human communities often co-exist and invasive species impacts are exacerbated^42,48^. Greater investment in invasive species eradications, particularly prioritized on larger islands and in Small Island Developing States, would make reliable gains towards restoring global biodiversity. The international transfer of knowledge for eradicating harmful invasive species from these islands is a key enabling condition that will strengthen eradication opportunities. Continuing this trend will require the support of national and international agreements alongside community empowerment and participation in invasive species eradication efforts^42,49^.

## Methods

The Database of Island Invasive Species Eradications (DIISE)^22^ collates the implementation of invasive vertebrate species eradication efforts on islands worldwide. An eradication operation is defined as the complete removal of a targeted invasive species from an entire island. Where multiple invasive species are eradicated from the same island, they are considered separate eradication events. Since its inception, the DIISE has undergone multiple systematic data collation processes, the most recent between 2017 and 2018 and finalized in 2019 with data made available from the 2017 International Conference on Island Invasives^50^. All DIISE data are publicly available in both Spanish and English at http://diise.islandconservation.org. Data definitions and systematic data collation methods are described elsewhere^13,20^.

### Summary of current database records

The DIISE presently contains 2166 eradication events on 1266 islands. These results were based on 1298 eradication resources, which included over 800 reports and published articles and 260 communications with experts. Following application of data quality and definition standards (described elsewhere^13^ and summarized below) the dataset for analysis comprised 1550 eradication events on 998 islands.

Despite utilizing data quality measures and expert review for assessing accuracy, we cannot rule out errors in the underlying dataset, which reflect the inherent trade-offs in summarizing these conservation interventions at the global scale. The true number of invasive species eradication events on islands will be greater than the number reported here for multiple reasons. First, publication of invasive species eradication attempts on islands remains relatively uncommon^51^ with results reported in this study often found in unpublished reports. Second, authors of this paper are first-language English speakers and while Google Translate was used to translate non-English reports, events may be overlooked due to this language bias^52^. Third, failures are an under-reported outcome in conservation, yet are essential for guiding conservation improvements^16,53^. Fourth, we expect a lag between an eradication being completed and reported and thus an underestimate of reported eradications in the late 2010s, and those planned for the 2020s. Given eradications are a temporal process with an evolving eradication status, the status of the most recent events may have already changed by the time of this publication (e.g., a planned event completed, or a reinvasion after previous successful eradication). Nonetheless, we expect this bias to be small and general success rates and trends reported here to be robust.

### Island characteristics

Island data, including unique island identification codes, location, area, name, and human habitation, follow the methodology described elsewhere^2^. Island area was recalculated in an equal area sinusoidal projection and presented in hectares. Country and territory names were based on International Standards Organization 3166-1 alpha-2 codes, with subsequent classification of Small Island Developing States^54^, and income levels (gross national income (GNI) per capita, in U.S. dollars^55^). Human habitation data was pooled into ordinal categories of 0, 1–10, 11–100, 101–1000, 1001–10,000, > 10,000, or *not found*.

### Criteria for data analysis

We used strict data quality standards for inclusion of data in this analysis (described elsewhere^13^), resulting in 1550 events. In summary, we only included eradication events where the (1) data quality status was good or satisfactory, (2) entire island was treated to achieve eradication (e.g., excluding restricted range eradications), (3) invasive populations were classified as feral or semi-feral (excluding domestic populations; e.g., farmed cattle), (4) the purpose was eradication (excluding eradication trials), and (5) eradication status was classified into one of seven categories: successful, successful and subsequently reinvaded, failed, to be confirmed, in progress, incomplete, and planned. Incomplete events are those that commenced but discontinued without attempting to complete the eradication, while in-progress events were still ongoing at the time of writing. Successful events are those where the target invasive species was declared absent from the treated island. Failed events are exclusively an operational failure, meaning that the operation did not succeed in completely removing the targeted invasive species from the island. This differs from a restoration failure whereby following a successful eradication the invasive species reinvaded and re-established on the island^56^. Only eradication statuses of successful, successful and subsequently reinvaded, to be confirmed, and failed were considered as completed operations (Supplementary Table S1). An end year was identified for each completed operation, defined as the last year that a method was applied. For hunting and trapping this is typically the same year the eradication outcome was declared, but confirmation of eradication success or failure for toxicant based operations does not occur when operations cease, but in years following (up to two years for rodents) during which target animals, if present, will breed to detectable levels^19^.

### Data analysis

We summarized invasive mammal species into nine groups based on a combination of their taxonomy, eradication strategy, and prevalence in the data (Table 1). Birds were divided into categories of flying and non-flying because eradication feasibility assessments and methods differ for this trait. For each of these invasive groups we summarized the 1550 events, enumerating events by status, success rates, island characteristics, and eradication methods (Table 1).

Success rate was based on events classified as either successful or failed only, excludeding successful events with a subsequent reinvasion, and was calculated as: success/[success + failure]. Success rate was calculated for invasive groups with at least 10 records or for years with at least five events. Success rates for reptile and amphibian eradications were not calculated; each class had fewer than 10 records and fewer than five efforts were attempted annually.

### Analyzing temporal eradication trends

We examined temporal trends in 1549 eradication events since 1900 (excluding the first eradication in 1872, which occurred nearly 40 years prior to the next eradication in 1911). There were 90 eradication events without an end date, with status classified as in progress, to be confirmed, or planned, and these were assigned the end date of 2019 for this analysis. While presuming a 2019 end date artificially inflated the number of events for that year, these events represented eradication events where effort was underway in scoping, planning, fundraising, and implementation. At the time of writing, those events may have started or completed (e.g., Seymour Norte). As such, we considered these 90 events as an important metric of contemporary activity, whereas excluding them may lead to a false impression of decline. We verified that the alternative strategy of excluding them made little difference to the overall curve trajectories, and that assigning events to 2019 was indeed the conservative option.

To observe temporal trends over time, we plotted four metrics in cumulative plots: eradication events, treated island area, countries implementing eradications, and invasive taxa targeted (Fig. 2, Supplementary Figs. 1, 2). To observe divergences in trends among eradication statuses, within the cumulative eradication events and targeted island area plots we compared (1) all events (includes all 7 status categories), (2) complete events (all events that were complete with one of the outcomes of successful, successful and subsequently reinvaded, or failed), and (3) successful events (completed events with a successful outcome).

To assess trends over time we also plotted the four metrics described above on an annual basis. We created a novel timeline analysis to quantify the trend shape for each metric and to compare timelines among the different metrics. For each annual plot we estimated a smooth trend curve using a cubic spline fit. Cubic spline curves have a convenient formulation that facilitates analysis of trend shape, which allowed us to identify time periods of significant growth or decline (i.e., “gradient”) and acceleration or deceleration (i.e., “curvature”) in each plot^57–59^.

A cubic spline is a smooth curve generated by a series of cubic polynomials joined at points known as knots^60^. We used generalized cross-validation^61^ to determine the number of equidistant knots for each curve, and assumed lognormal scatter of the data about the curve. We assessed precision of the estimated trend curve by using 1000 replicates of the parametric bootstrap to generate pointwise 95% confidence intervals. For the area metric, there was a sharp change in lognormal variance in the mid-1980s, so the scatter model was applied piecewise for this curve.

We quantified the shape of the estimated trend curves by computing the gradient (rate of change per year) and curvature (change in gradient per year) trajectories over time. A positive curvature indicates a period of acceleration where the trend curve turns upwards, implying an improvement in growth rate from one year to the next, and a negative curvature indicates a period of deceleration where the trend curve turns downwards. Curvature values near zero describe a trajectory that is roughly linear, signifying constant growth or decline.

The gradient and curvature trajectories were computed for each of the bootstrapped spline curves using the first and second derivatives of the curves, yielding pointwise 95% confidence intervals for each shape attribute. The gradient or curvature was statistically significant at times when the corresponding 95% confidence interval did not contain the value zero. These periods can be interpreted as times of significant growth or acceleration for positive gradient or curvature, respectively, and as times of significant decline or deceleration for negative gradient or curvature^57^. For both shape attributes we identified periods where a pattern was consistently significant for at least 5 consecutive years. All analyses were performed using the R statistical environment (version 4.1.1^62^).

## Supplementary Information

Supplementary Information 1.

Supplementary Information 2.

## Data Availability

The Database of Island Invasive Species Eradications can be found and downloaded at http://diise.islandconservation.org. Data from the database and the code used in this analysis are available via the Zenodo Digital Repository at 10.5281/zenodo.6663551. Data quality standards and recommendations for data usage are described in this article; further guidance is available upon request.
