# Supplementary material for: The global contribution of invasive vertebrate eradication as a key island restoration tool
Source: Sci Rep. 2022 Aug 10;12:13391. doi: 10.1038/s41598-022-14982-5 (PMC9365850; doi:10.1038/s41598-022-14982-5)
Supplement: Supplementary file 1 — Supplementary Information 1. [file 41598_2022_14982_MOESM1_ESM.pdf]

Supplementary Information

Title: The global contribution of invasive vertebrate eradication as a key island restoration tool

Authors: Dena R. Spatz[1]*, Nick D. Holmes[2], David J. Will[3], Stella Hein[3,8], Zachary T. Carter[4], Rachel M. Fewster[4], Brad Keitt[5], Piero Genovesi[6], Araceli Samaniego[7], Donald A. Croll[8], Bernie R. Tershy[8] & James C. Russell[4]

This document contains:

- Supplementary Table S1*:* Invasive vertebrate eradication targets and eradication status reported for 1550 events,1872 - 2019.
- Supplementary Figures 1 - 4
- Supplementary Discussion

Supplementary Table S1. Invasive vertebrate eradication targets and eradication status reported for 1550 events,1872 - 2019. Completed events include the event status of successful, successful and subsequently reinvaded, or failed. Ongoing events include the event status of in progress or to be confirmed.

**Invasive target groups**	Completed Events	Incomplete Events	Ongoing Events	Planned Events	Total # Events
Rats (*Rattus* spp.)	754	1	39	26	820
Ungulates	167	9	6	3	185
Cats	97	4	7	9	117
House Mice (*Mus musculus)*	77	6	0	8	91
Rabbits & Hares	82	5	1	1	89
Dogs & Foxes	68	0	1	0	69
Stoats, Weasels, Mink & Mongoose	58	0	7	1	66
Other mammals	30	1	5	3	39
Pigs	27	1	0	2	30
Flying birds	17	2	4	1	24
Non-flying birds	14	0	1	0	15
Frogs & Toads	2	1	1	0	4
Lizards	1	0	0	0	1
**TOTALS**	1394	30	72	54	1550

Supplementary Figure 1. Eradication events by invasive vertebrate taxa, 1900 – 2019. Rats (*Rattus spp.*) y-axis scaled to 800 eradication events due to high eradication event totals, remaining panels scale to 200 events.

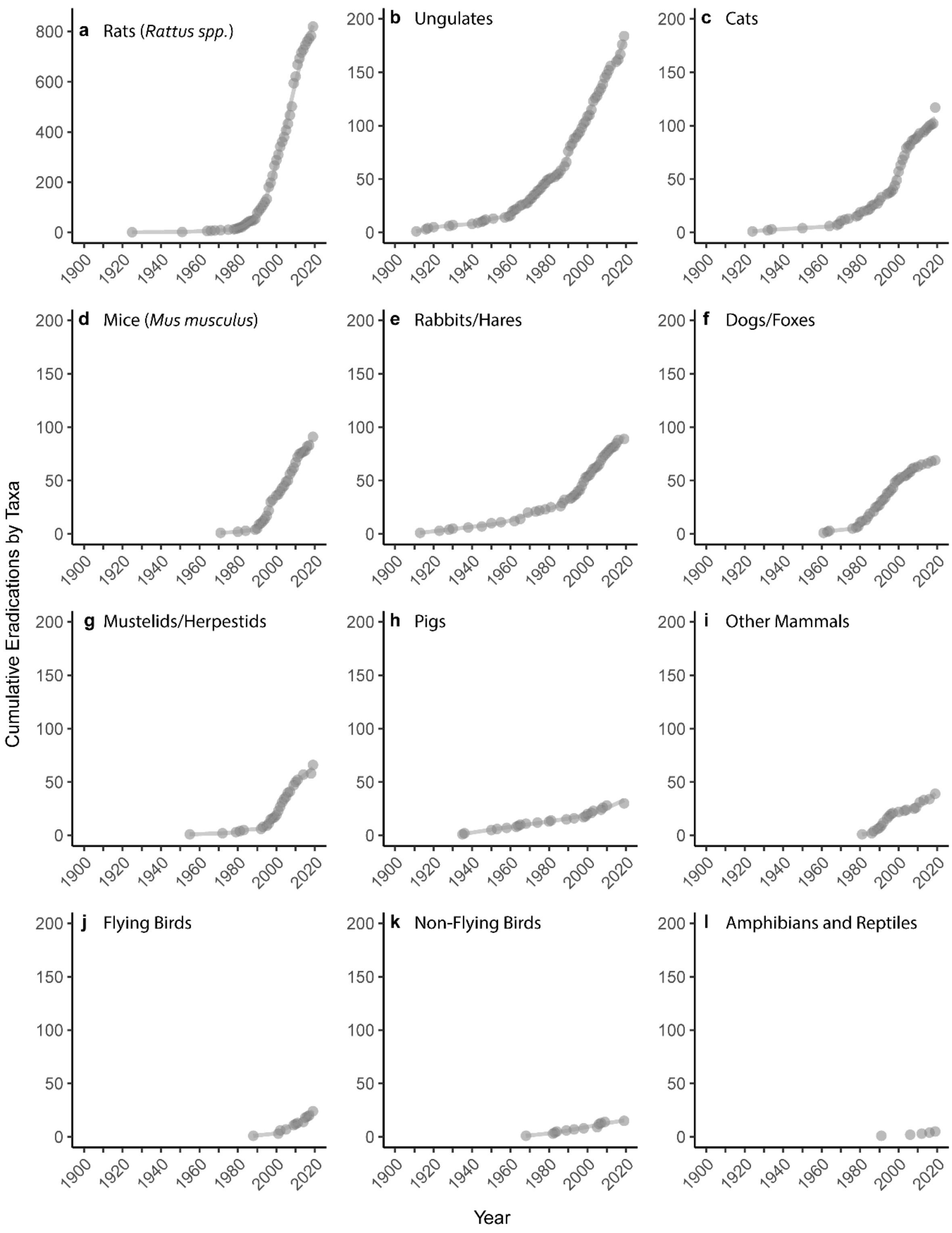

Supplementary Figure 2. Eradication events by implementing country, distinguishing the top eight countries by frequency, 1900 – 2019.

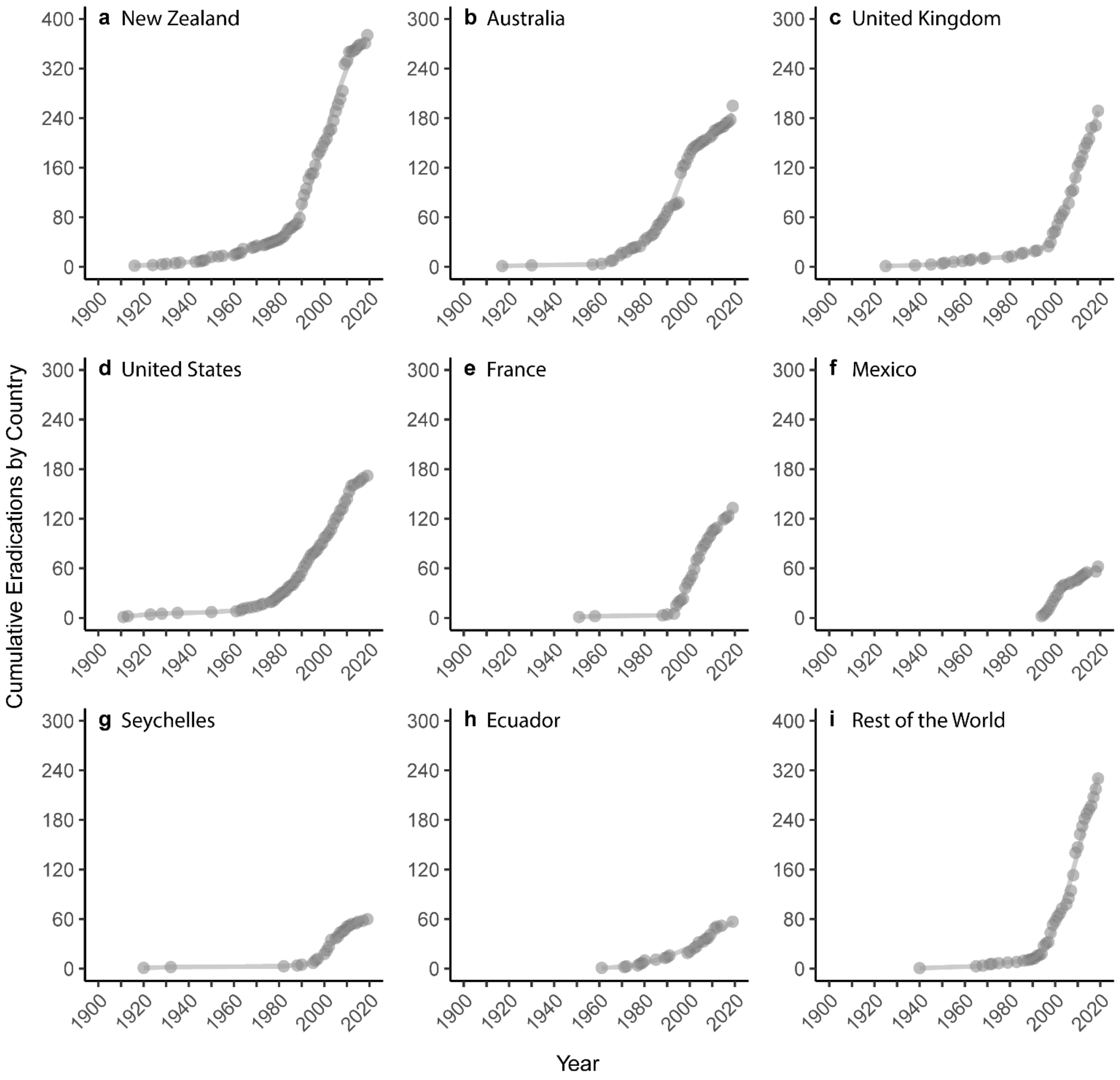

Supplementary Figure 3. Treated island area (ha), log scale, 1900 – 2019.

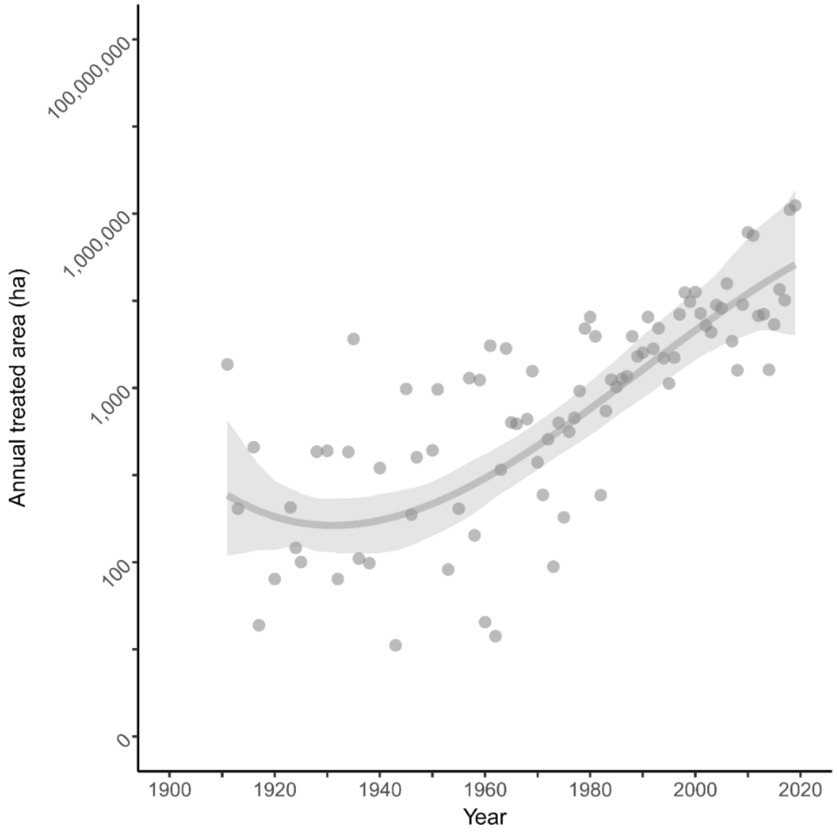

Supplementary Figure 4. Treated island area by taxa, 1900 – 2019.

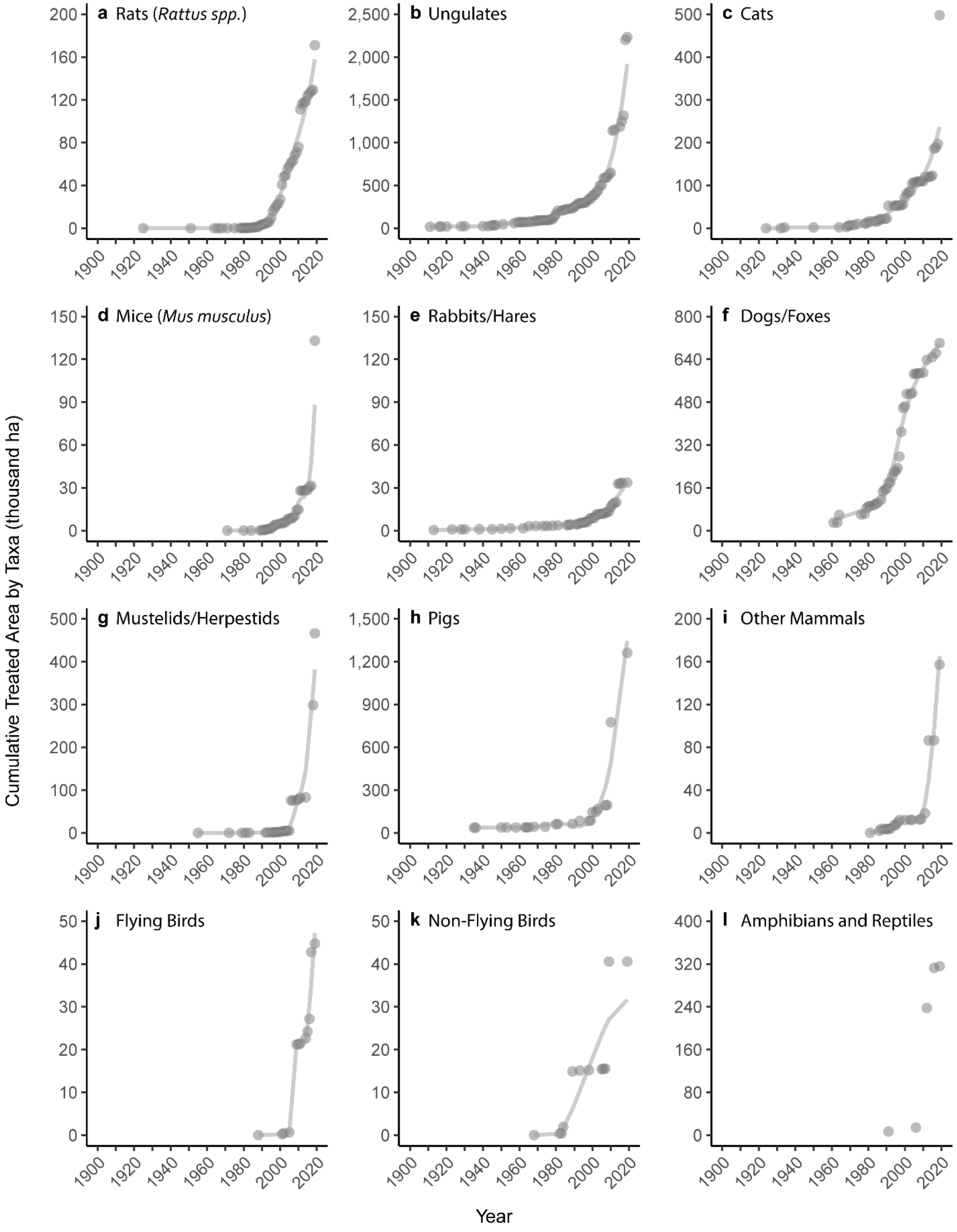

Supplementary Discussion

Excluded data and notable eradication events

A total of 2166 eradication events were documented in the database, of which 449 were excluded because of poor data quality. An additional 167 events were removed because the activity did not aim to remove an invasive wild animal from an entire island, the activity was a trial or had an unknown outcome, and/or the activity did not treat the entire island. Of those that did not treat an entire island, 59 were incursion responses and 49 were restricted range eradications. These activities do ultimately clear an island of an invasive species, but the invasive species is only targeted on part of an island, making these activities operationally different from and statistically unrepresentative as “whole island” eradications. However, notable restricted range eradication efforts do include the successful rat, mouse, and reindeer eradications from the 3,500 km^2 South Georgia Island and the failed removal of cats and rabbits from the 6,715 km^2 Grande Terre Island. These projects play important roles in improving practitioner knowledge regarding invasive vertebrate eradication “restricted range” techniques, particularly on these relatively large islands.

A brief summary of invasive mouse eradication successes and failures

Invasive rodent eradications include rats (*Rattus*) with an 88% success rate, and mice (*Mus musculus*) with a 73% success rate, the latter representing the lowest among all eradication targets analyzed. This low success rate occurred during early rodent eradication efforts when mouse eradications were initially attempted concurrently with eradications for the larger bodied rat species, typically the primary target given impact[1,2]. However, it is now known that these

operational designs may not have catered to the ecology of mice, including differences in food preferences and smaller home ranges compared to rats[2]. In New Zealand, when these factors were considered and changes in operational techniques were subsequently undertaken, mouse eradication success rate was >90%[3]. Since the mid-2000s mouse eradication success rates globally have dramatically improved thanks to developed best practice techniques (Supplementary Table 1) [1].

A brief summary of invasive avian eradications

This study primarily focuses on mammal eradications as they make up over 97% of invasive vertebrate eradication activities on islands. However, other invasive vertebrate taxa have been targeted for eradication. Notably, 64 eradication attempts of invasive birds occurred since the 1960s, primarily since the 2000s (n=31, 48%), with an 83.7% success rate. Sixty-two percent of these invasive bird eradications targeted *Acridotheres tristis* (common myna) and *Gallirallus australis* (weka). However, 23 of these events (36%) were removed from our analysis because they were either incursion responses (n=5) or restricted range activities (n=18). Invasive birds are a major threat on islands[4], with particularly negative impacts on agriculture, and hence food security for human communities[5,6]. hence, the ongoing removal of invasive bird populations from islands could be an important means of progressing towards both biodiversity and sustainable development goals[7,8].

Supplementary Discussion References

1. Samaniego, A. *et al.* Factors leading to successful island rodent eradications following initial failure. *Conserv. Sci. Pract.* **3**, 1–12 (2021).
2. MacKay, J. W. B., Russell, J. C. & Murphy, E. C. Eradicating house mice from islands: successes, failures, and the way forward. *Manag. Vertebr. invasive species Proc. an Int. Symp.* **1971**, 294–304 (2007).

3. Broome, K. *et al.* House mice on islands: management and lessons from New Zealand. *Isl. invasives scaling up to meet challenge.* 100–107 (2019).
4. Evans, T., Kumschick, S., Şekercioğlu, C. & Blackburn, T. Identifying the factors that determine the severity and type of alien bird impacts.pdf. *Biodivers. Res.* **24**, 800–810 (2018).
5. Runde, D. E., Pitt, W. C. & Foster, J. T. Population ecology and some potential impacts of emerging populations of exotic parrots. *Manag. Vertebr. Invasive Species. Pap. 42.* 338–360 (2007).
6. Shiels, A. B. & Kalodimos, N. P. Biology and Impacts of Pacific Island Invasive Species. 15. Psittacula krameri, the Rose-Ringed Parakeet (Psittaciformes: Psittacidae). *Pacific Sci.* **73**, 421 (2019).
7. United Nations. Transforming our world: the 2030 Agenda for Sustainable Development. in *Follow- up to the outcome of the Millennium Summit (A/69/L8.5)* (UN General Assembly, 2015).
8. United Nations. The Strategic Plan for Biodiversity 2011-2020 and the Aichi Biodiversity Targets. in *Conference of the Parties to the Convention on Biological Diversity, 10* (2010).
